# Online and Offline Social Sensitivity as Mediator Between Online Vigilance and Depression, Anxiety, and Stress Among Algerian Female Students

**DOI:** 10.1002/pchj.70017

**Published:** 2025-05-13

**Authors:** Aiche Sabah, Ahmed Alduais, Musheer A. Aljaberi, Mahshid Manouchehri

**Affiliations:** ^1^ Faculty of Human and Social Sciences Hassiba Benbouali University of Chlef Chlef Algeria; ^2^ Department of Psychology Norwegian University of Science and Technology Trondheim Norway; ^3^ Department of Internal Medicine, Section Nursing Science Erasmus University Medical Center (Erasmus MC) Rotterdam the Netherlands; ^4^ Research Centre Innovations in Care Rotterdam University of Applied Sciences Rotterdam the Netherlands; ^5^ Amity University Dubai Campus Dubai UAE

**Keywords:** Algerian female students, mental health outcomes, online vigilance, psychological distress, social sensitivity

## Abstract

The purpose of this study is to investigate the mediating role of online and offline social sensitivity in the association between online vigilance and mental health outcomes—specifically depression, anxiety, and stress—among Algerian female university students. A cross‐sectional study was conducted with 438 Algerian female university students. Validated scales were utilized to measure online vigilance, social sensitivity (both online and offline), and psychological distress. Data was analyzed using correlation and mediation analyses to explore the relationships among these variables and to assess the mediating effect of social sensitivity. The findings indicate a significant positive relationship between online vigilance, social sensitivity, and mental health problems. Online and offline social sensitivity fully mediated the influence of online vigilance on stress, anxiety, and depression. This suggests that higher engagement in online vigilance increases social sensitivity, which in turn heightens vulnerability to psychological distress. This study contributes to the understanding of the complex interplay between digital behaviors, social perceptions, and mental health among Algerian female university students. By highlighting the mediating role of social sensitivity, the research underscores the necessity for interventions that address online habits and enhance social coping skills to improve mental well‐being in this population.

## 1 Introduction

The swift progression of digital technologies and social media channels has profoundly altered the dynamics of human interaction and communication modalities in modern society (Carr and Hayes 2015; Hampton 2016; Levin and Mamlok 2021). Although these advancements provide extraordinary global connectivity and ease of use (Shanmugasundaram and Tamilarasu 2023), they simultaneously pose challenges to conventional face‐to‐face interactions and evoke apprehensions regarding privacy and the transformation of communication standards (Amelia and Balqis 2023). The integration of digital technologies and social media into everyday life has obscured the distinctions between public and private domains, created novel modalities of digital communication, and altered the character of interpersonal relationships (Anista 2023).

Internet usage in high‐income countries increased from 90.5% in 2023 to 91.6% in 2024, while low‐income countries increased from 20.9% to 23.6%. In lower middle‐income economies like Algeria, internet utilization increased from 56.3% to 59.0% (Union 2024). Social media usage has been increasing worldwide and is expected to reach high percentages by 2025. Facebook would reach 10 billion users, LinkedIn 1.5 billion, and Twitter 750 million (Jayaram et al. 2020). It fundamentally alters human behavior, including outrage, status‐seeking, and mental health (Van Bavel et al. 2024). According to a GSMA report on mobile internet usage (GSMA 2024), the gender gap is narrowing in low‐ and middle‐income countries. In 2023, 66% of women and 78% of men use mobile internet, with women 15% less likely than men to use mobile internet. Women and men reported ownership at 60% and 69%, respectively. Women's adoption of mobile internet in the past year has increased at a higher rate than that of men, which is a positive shift toward digital inclusion. The most frequent uses of smartphones among women and men are social media and instant messaging. However, women are more inclined to continue increasing that use in the future, meaning there is future potential in engaging more females with mobile technologies.

Internet use among female students is intricately linked to online vigilance, which refers to a heightened awareness of online interactions and communication (Van Gaeveren et al. 2024). Research shows that female students who exhibit higher levels of online vigilance demonstrate better academic self‐control, particularly in perseverance and attention. This relationship is significant, as academic attention and various dimensions of online vigilance–such as salience and reactivity–are positively correlated in female students, suggesting that their engagement with digital platforms enhances their focus on academic tasks (Kıdıman and Durak 2023). However, the implications of online vigilance extend to mental health, where studies indicate that while online vigilance may not pose serious threats to affective well‐being, it can contribute to increased stress and anxiety due to the pressures of maintaining an active online presence (Johannes et al. 2021). Furthermore, the constant need for connectivity can affect social sensitivity, potentially leading to both positive and negative outcomes in mental health (Karim et al. 2020). Thus, understanding the dynamics of online vigilance among female students is essential for addressing their social sensitivity and its potential effects on depression, anxiety, and stress.

### 1.1 Online Vigilance

Online vigilance, the constant preoccupation with online communication, encompasses three dimensions: salience (mental preoccupation with online interactions), reactibility (responsiveness to digital stimuli), and monitoring (frequency of unprompted device checks) (Johannes et al. 2021; Reinecke et al. 2018). Among students, this behavior has become increasingly common due to smartphones and social media, impacting academic performance and behavior. Research shows a weak negative association between online vigilance and academic performance, with media use and online vigilance predicting 9% of the variance in academic performance (Le Roux et al. 2021). Gender differences exist, with academic attention significantly related to all sub‐dimensions of online vigilance in females but not in males (Kıdıman and Durak 2023). Boredom proneness predicts online vigilance behaviors like phubbing (Lv and Wang 2023), and online vigilance fully mediates the relationship between excessive media use in class and academic performance (Yin et al. 2024). While online vigilance can provide social support and gratification (Johannes 2020), it also leads to decreased mindfulness and increased mind‐wandering, negatively impacting life satisfaction and affective well‐being (Johannes et al. 2018). The salience dimension of online vigilance is particularly detrimental to affective well‐being, with the valence of thoughts being more crucial than their frequency (Johannes et al. 2021). These findings suggest that the impact of online vigilance on well‐being may vary depending on individual factors and specific dimensions of vigilance.

### 1.2 Online and Offline Social Sensitivity

The concept of Online and Offline Social Sensitivity refers to the degree to which individuals are sensitive to social rejection in both digital and face‐to‐face interactions. This sensitivity is crucial because social rejection, whether online or offline, can significantly impact an individual's psychological well‐being (Andrews et al. 2022). The Online and Offline Social Sensitivity concept was developed to measure variations in sensitivity to social cues across these contexts, providing valuable insights into the psychological effects of living in a digital age (Reinecke et al. 2017). Females may exhibit higher levels of social sensitivity both online and offline. This increased sensitivity is often due to sociocultural factors that emphasize the importance of social relationships and acceptance in women's lives. Studies have shown that females are more likely to experience and report feelings of social rejection and exclusion, which can be intensified by the pervasive nature of social media and online interactions (Andrews et al. 2022). The relationship between Online and Offline Social Sensitivity and mental health is significant. High levels of social sensitivity can lead to increased vulnerability to mental health issues such as depression, anxiety, and low self‐esteem. Frequent perceptions of social rejection, whether online or offline, contribute to negative emotional states and cognitive patterns like rumination and brooding, underscoring the need to address social sensitivity to promote psychological resilience (Andrews et al. 2022). Research indicates that social rejection sensitivity in both contexts is a risk factor for depression, especially among young people, and is associated with higher levels of depressive symptoms and ruminative brooding (Andrews et al. 2022).

### 1.3 Mental Health in the Digital Age

The digital age has significantly impacted mental health, particularly among females, due to the widespread use of digital devices and social media platforms. Research by Joshi et al. (2021) found that excessive use of digital devices among university students in Nepal is linked to high levels of depression, anxiety, and loneliness. Similarly, Al Salman et al. (2020) reported high rates of anxiety and depression among female secondary school students in Al‐Khobar, Saudi Arabia, due to excessive electronic device use. Ghaemi (2020) highlighted the rise in depression, anxiety, and suicidality among teenagers and young adults with the advent of smartphones and social media, emphasizing the need for clinical guidelines to limit social media use. Lukenga et al. (2023) suggested that digital tools and biofeedback could help female students manage academic stress more effectively. In conclusion, addressing the mental health challenges posed by excessive digital device use requires targeted interventions, awareness programs, and the integration of digital resilience and positive online behaviors into educational systems.

### 1.4 Role of Online and Offline Social Sensitivity as Mediator

The role of online and offline social sensitivity as mediators is essential in understanding the psychological effects of online vigilance on emotional well‐being. Social rejection sensitivity (SRS) plays a crucial part in this mediation, as adolescents with high SRS are more prone to emotional disorder symptoms and negative interpretation biases (Minihan et al. 2023). This sensitivity can exacerbate the impact of online vigilance, leading to problematic internet use (Águila Rodríguez 2023). High levels of online mindfulness increase individuals' sensitivity to social cues, resulting in heightened anxiety and stress during online interactions (Reinecke et al. 2017). However, offline social sensitivity can mitigate these negative effects by offering supportive and meaningful social interactions, reducing symptoms of depression, anxiety, and stress (Seabrook et al. 2016). Therefore, promoting healthy social sensitivity and effectively managing online vigilance can help decrease the cognitive and emotional burdens associated with digital interactions, fostering better psychological health.

### 1.5 Current Study

The profound social, economic, and political transformations within Algerian society have reshaped family dynamics and daily life (Sabah, Aljaberi, Hajji, et al. 2024; Sabah, Aljaberi, Hamouda, et al. 2024; Sabah, Aljaberi, Lee, et al. 2023; Sabah, Aljaberi, Hajji, et al. 2023; Sabah et al. 2022). These changes have accelerated the integration of technology, particularly the internet, into social interactions, with families and individuals increasingly relying on digital platforms to navigate these societal shifts (Sabah, Aljaberi, Lee, et al. 2023; Sabah, Aljaberi, Hajji, et al. 2023). Consequently, internet use has surged, creating new forms of social engagement and sensitivity, especially among Algerian female students, who now experience heightened online vigilance in their day‐to‐day interactions.

While much research has been conducted on online vigilance, social sensitivity, and mental health in various global contexts, few studies have specifically explored these dynamics among female students in the Algerian context, where cultural norms, societal expectations, and the rapid technological transformation play crucial roles in shaping these experiences. Moreover, the existing literature largely focuses on the general population's social sensitivity and online behavior without examining the potential mediating role of online and offline social sensitivity between online vigilance and mental health outcomes such as depression, anxiety, and stress. Given these gaps, this study aims to address the following research question: Do online and offline social sensitivity mediate the indirect effects of online vigilance on stress, anxiety, and depression among Algerian female students?

### 1.6 Research Hypothesis

The study hypothesizes that Online Offline Social Sensitivity mediates the relationship between Online Vigilance and psychological distress (stress, anxiety, and depression). Specifically, it posits that higher levels of Online Vigilance lead to increased Online Offline Social Sensitivity, which in turn results in greater levels of stress, anxiety, and depression.

## 2 Methods

### 2.1 Participants

A cross‐sectional design was used in the current study. The electronic application was conducted through Google Forms, where the study tools were applied to 438 female university students in Algeria using the snowball sampling method. The link containing the questionnaires was electronically distributed to the students and then forwarded to professors from various Algerian universities, who were also asked to provide the link to the students for completion. The study was conducted during the year 2024. Missing data was controlled by enabling the feature that prevents submission unless all answers are filled out. The exclusion criteria included male students and those who had completed their university education, while the inclusion criteria were female students currently enrolled in university.

The demographic characteristics of female university students, as shown in Table 1, reveal a predominantly young and single population, with 77.9% aged between 18 and 23 years and 88.1% being single. The majority are in their second (41.6%) and third (30.8%) years of undergraduate studies, with a notable presence of graduate students, including 17.1% in their second year of master's programs and 1.8% pursuing PhDs. A significant concentration of students (74.9%) is majoring in Humanities and Social Sciences.

**TABLE 1:** Demographic characteristics of the participants.

Variables		Frequency	Percentage
Age	18–23 years	341	77.9
	24–30 years	39	8.9
	31–40 years	41	9.4
	41–50 years	16	3.7
	51–60 years	1	0.2
Marital status	Single	386	88.1
	Married	49	11.2
	Divorced	3	0.7
Education level	First year undergraduate	14	3.2
	Second year undergraduate	182	41.6
	Third year undergraduate	135	30.8
	First year Master's	24	5.5
	Second year Master's	75	17.1
	PhD	8	1.8
Major	Medicine and Surgery	1	0.2
	Engineering (Civil, Electrical, Mechanical)	1	0.2
	Computer Science and Information Technology	3	0.7
	Economics and Business	2	0.5
	Natural Sciences (Chemistry, Physics, Biology)	6	1.4
	Agriculture and Food Sciences	1	0.2
	Humanities and Social Sciences (Literature, Languages, Sociology, Psychology, Sports)	328	74.9
	Law and Political Science	4	0.9
	Journalism and Media	5	1.1
	Other	87	19.9

### 2.2 Instruments

#### 2.2.1 Online Vigilance Scale

The study utilized the Online Vigilance Scale developed by Reinecke et al. (2018) to measure online vigilance, which encompasses Salience, Reactibility, and Monitoring constructs. This scale effectively captures individuals' awareness, emotional responses, and active monitoring of online content. The 12‐item scale demonstrates strong psychometric properties, including high internal consistency (Cronbach's α values: 0.91 for Salience, 0.83 for Reactibility, and 0.91 for Monitoring) and a valid factor structure confirmed by confirmatory factor analysis (Reinecke et al. 2018). In the current study, Cronbach's alpha was 0.88, and McDonald's ω was 0.88.

#### 2.2.2 The Online and Offline Social Sensitivity Scale

The Online and Offline Social Sensitivity Scale (O2S3), developed by Andrews et al. (2022), assesses individuals' sensitivity to social rejection in both digital and physical settings. Comprising 18 items evaluated on a four‐point Likert scale, it measures four factors: Concern about Approval, Online Social Interaction, Social Commitment, and Social Risk Taking. Higher scores reflect greater social sensitivity. Confirmatory factor analyses and reliability tests (Cronbach's alpha = 0.88) demonstrated the scale's validity and consistency, making it a reliable tool for measuring social rejection sensitivity. Cronbach's alpha coefficient in the current study was estimated at 0.82.

#### 2.2.3 Depression Anxiety Stress Scale‐21 (DASS‐21)

The Depression Anxiety Stress Scale‐21 (DASS‐21) was developed by (Lovibond and Lovibond 1995). It consists of three main dimensions: Depression, Anxiety, and Stress. DASS‐21 comprises 21 items, where participants rate their state on a scale from 0 to 3 (Never, Sometimes, Often, Always) for each item. The total score is calculated by summing the points of the seven items in each dimension, and the total score is divided into categories representing the levels of depression, anxiety, and stress. The study by (Mohammed 2021) verified the validity and reliability of the DASS‐21 scale, with a Cronbach's alpha coefficient of 0.91, indicating high internal consistency. Split‐half reliability was also used to assess the stability and reliability of the scale, showing good quality for the measure. In the current study, Cronbach's alpha reached 0.90.

#### 2.2.4 Data Analysis

For statistical analysis, descriptive statistics were conducted using IBM SPSS Statistics Version 26, while mediation analysis was performed using JASP. Measurement models and structural models were evaluated to analyze complex behavioral relationships (Sabah et al., 2024; Sabah et al. 2021). The structural model employed in the study used maximum likelihood estimation to explore the structural model connecting Online Vigilance as the independent variable, with stress, anxiety, and depression as dependent variables, and Online‐Offline Social Sensitivity as the mediating variable. Additionally, the bootstrapping technique in JASP (with 1000 bootstrap samples) was used to test and confirm the mediation hypothesis.

## 3 Results

### 3.1 Preliminary Analysis

Table 2 provides a detailed overview of the descriptive statistics and correlation coefficients for the key variables under investigation: Online Vigilance, Online–Offline Social Sensitivity, Stress, Anxiety, and Depression. Means (M), standard deviations (SD), skewness, and kurtosis are reported for each variable to offer insight into their distributional properties. Online Vigilance had M = 33.3 (SD = 9.8), skewness = −0.04, and kurtosis = −0.24, indicating a relatively symmetric distribution. Online–Offline Social Sensitivity had M = 37.6 (SD = 7.5), skewness = −0.05, and kurtosis = 0.06, also suggesting a near‐normal distribution. Stress had M = 10.9 (SD = 4.0), skewness = −0.22, and kurtosis = −0.10, reflecting a slight deviation from normality. Anxiety had M = 11.1 (SD = 5.5), skewness = −0.07, and kurtosis = −0.68, and Depression had M = 9.4 (SD = 6.0), skewness = 0.44, and kurtosis = −0.53. These skewness and kurtosis values indicate acceptable deviations from normality, making parametric analyses appropriate.

**TABLE 2:** Descriptive statistics and correlations among study variables.

Variables	M	SD	Skewness	Kurtosis	R				
					1	2	3	4	5
1. Online Vigilance	33.3105	9.82009	−0.039	−0.235	—				
2. Online Offline Social Sensitivity	37.5982	7.51189	−0.048	0.062	0.465**	—			
3. Stress	10.8493	4.02795	−0.217	−0.101	0.157**	0.205**	—		
4. Anxiety	11.1347	5.54141	−0.065	−0.680	0.170**	0.278**	0.569**	—	
5. Depression	9.4256	6.03940	0.436	−0.532	0.220**	0.326**	0.534**	0.509**	—

** *p* < 0.001.

The correlation matrix revealed significant relationships among the variables. Online Vigilance was significantly correlated with Online–Offline Social Sensitivity (*r* = 0.47, *p* < 0.001), suggesting that individuals more vigilant online also tend to exhibit higher social sensitivity across contexts. Additionally, Online–Offline Social Sensitivity was positively correlated with Stress (*r* = 0.21, *p* < 0.001), Anxiety (*r* = 0.28, *p* < 0.001), and Depression (*r* = 0.33, *p* < 0.001), indicating that heightened social sensitivity is associated with increased psychological distress. Stress, Anxiety, and Depression exhibited high intercorrelations: Stress correlated with Anxiety (*r* = 0.57, *p* < 0.001) and Depression (*r* = 0.53, *p* < 0.001), and Anxiety correlated with Depression (*r* = 0.51, *p* < 0.001).

### 3.2 Mediation Analysis

#### 3.2.1 Direct Effects of Online Vigilance on Stress, Anxiety, and Depression

The direct effect of Online Vigilance on stress is positive but not statistically significant (*p* = 0.13) (See Table 3). The confidence interval (−0.003 to 0.021) includes zero, indicating that this effect is nonsignificant. Similarly, the direct effect of Online Vigilance on anxiety is also positive but not statistically significant (*p* = 0.32). The estimate of 0.005 indicates a small positive effect, but the confidence interval (−0.005 to 0.017) again crosses zero, suggesting that the effect is not significant. This indicates that Online Vigilance may not have a direct impact on anxiety levels. The direct effect of Online Vigilance on depression is nonsignificant (*p* = 0.09), with an estimate of 0.009. However, the confidence interval (−0.001 to 0.019) still includes zero, indicating that this effect is not statistically conclusive at the conventional 0.05 significance level.

**TABLE 3:** Direct effects of online vigilance on stress, anxiety, and depression.

					95% Confidence Interval	
Direct effects	Estimate	Std. error	z‐value	*p*	Lower	Upper
Online_Vigilance → Stress	0.008	0.005	1.499	0.134	−0.003	0.021
Online_Vigilance → Anxiety	0.005	0.005	1.005	0.315	−0.005	0.017
Online_Vigilance → Depression	0.009	0.005	1.696	0.090	−0.001	0.019

*Note:* Delta method standard errors, bias‐corrected percentile bootstrap confidence intervals, ML estimator.

#### 3.2.2 Indirect Effects of Online Vigilance on Stress, Anxiety, and Depression via Online Offline Social Sensitivity

The indirect effect of Online Vigilance on stress through Online Offline Social Sensitivity is statistically significant (*p* = 0.002), with an estimate of 0.008 (See Table 4). The confidence interval (0.002 to 0.014) does not include zero, indicating a robust mediation effect. This suggests that increased Online Vigilance leads to higher Online Offline Social Sensitivity, which in turn results in elevated stress levels.

**TABLE 4:** Indirect effects of online vigilance on stress, anxiety, and depression.

Indirect effects						
					95% Confidence Interval	
	Estimate	Std. error	z‐value	*p*	Lower	Upper
Online Vigilance → Online Offline Social Sensitivity → Stress	0.008	0.003	3.060	0.002	0.002	0.014
Online Vigilance → Online Offline Social Sensitivity → Anxiety	0.012	0.003	4.476	< 0.001	0.007	0.018
Online Vigilance → Online Offline Social_Sensitivity → Depression	0.013	0.003	4.980	< 0.001	0.009	0.019

*Note:* Delta method standard errors, bias‐corrected percentile bootstrap confidence intervals, ML estimators.

The indirect effect of Online Vigilance on anxiety via Online Offline Social Sensitivity is highly significant (*p* < 0.001), with an estimate of 0.012. The confidence interval (0.007 to 0.018) confirms the significance of this mediation effect. This indicates that individuals with higher levels of Online Vigilance experience greater Online Offline Social Sensitivity, which subsequently increases their anxiety levels.

Similarly, the indirect effect of Online Vigilance on depression through Online Offline Social Sensitivity is also highly significant (*p* < 0.001), with an estimate of 0.013. The confidence interval (0.009 to 0.019) further supports the presence of a significant mediation effect. This finding implies that higher Online Vigilance enhances Online Offline Social Sensitivity, leading to increased depressive symptoms.

#### 3.2.3 Total Effects of Online Vigilance on Stress, Anxiety, and Depression

**FIGURE 1:**
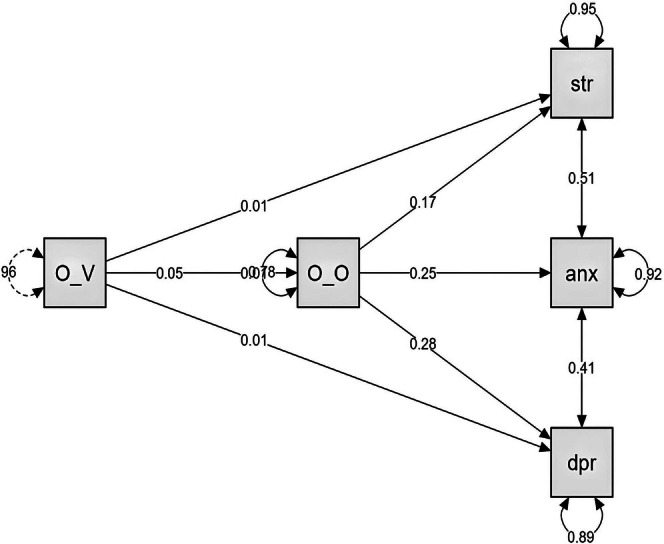
Path plot for online vigilance (OV) and online‐offline online and offline social sensitivity (OO) and stress (str), anxiety (anx), and depression (dpr).

The total effect of Online Vigilance on stress is statistically significant (*p* < 0.001), with an estimate of 0.02 (See Table 5 and Figure 1). The confidence interval (0.01 to 0.03) does not include zero, indicating a robust effect. The total effect of Online Vigilance on anxiety is also significant (*p* < 0.001), with an estimate of 0.02. The confidence interval (0.01 to 0.03) confirms the significance of this effect. The total effect of Online Vigilance on depression is particularly pronounced (*p* < 0.001), with an estimate of 0.02. The confidence interval (0.01 to 0.03) further supports the significance of this effect.

**TABLE 5:** Total effects for online vigilance and stress, anxiety, and depression.

Total effects						
					95% Confidence Interval	
	Estimate	Std. error	z‐value	*p*	Lower	Upper
Online_Vigilance → Stress	0.016	0.005	3.325	< 0.001	0.006	0.027
Online_Vigilance → Anxiety	0.017	0.005	3.609	< 0.001	0.008	0.027
Online_Vigilance → Depression	0.022	0.005	4.681	< 0.001	0.012	0.032

*Note:* Delta method standard errors, bias‐corrected percentile bootstrap confidence intervals, ML estimator.

The mediation analysis revealed full mediation of Online Offline Social Sensitivity in the relationship between Online Vigilance and psychological distress (stress, anxiety, and depression) among female students. These findings underscore the differential impacts of Online Offline Social Sensitivity and Online Vigilance on psychological states, highlighting their distinct roles in influencing stress, anxiety, and depression.

## 4 Discussion

The research investigated the mediating role of online and offline social sensitivity within the relationship between online vigilance and psychological distress, in terms of stress, anxiety, and depression, among female Algerian university students. These results evidenced that online vigilance significantly affects psychological distress indirectly through heightened social sensitivity, providing evidence of the complex interaction of digital behaviors and social‐cognitive factors in the etiology of mental health disturbances.

Results insinuate that socially sensitive female students from Algeria are more prone to manifesting forms of distress within online and offline contexts. Typically, the individual is acutely sensitive to social events, mainly those of acceptance/rejection, and shows hypersensitivity to perceived criticism or exclusion (Andrews et al. 2022). This accords with previous findings where socially sensitive people become prone to anxiety and depression in the face of negative social feedback, especially in ambiguous or, worse, hostile online interaction forums (Minihan et al. 2023).

Algerian women students could therefore also develop greater social sensitivity through the cultural aspects of family, community bonding, and social standing. All social harmony and upholding one's image are very important within Algerian society, especially when it comes to women. Such social pressure enhances the emotional impact of online interactions—rejection or exclusion could be felt not only as a personal failure but also as one reflecting on the family. For socially sensitive individuals, online vigilance thus becomes a source of psychological distress because they will need to surveil and manage social interactions so as not to be faced with judgments.

Socially sensitive individuals have often been found to have high levels of social rejection sensitivity, leading them further down the line into interpreting ambiguous social interactions in negative ways. As several studies have shown, the more sensitive a female is about social signs, the greater her risk for anxiety and depression. Online vigilance, in this case, is quite significant because continuous monitoring of online communications increases one's exposure to possible rejection, furthering stress and anxiety. In fact, according to Johannes et al. (2021), this would be especially the case with online vigilance.

Our results therefore indicated that online vigilance enhances social sensitivity, especially online. Reinecke et al. (2017) note that individuals who are more sensitive online and watchful can be highly sensitive to received social feedback; therefore, they may easily become emotionally unstable during their online communication. The Algerian culture views one's reputation and social relations as important; it would therefore make the students from the Middle East and the Arab region more vulnerable due to pressure in achieving a high status both online and offline.

This buffers the impact of negative online social sensitivity. Another mitigating factor is offline social sensitivity. Seabrook et al. (2016) reported that great face‐to‐face interactions can serve as a buffer against the negative impacts of heightened online social sensitivity. Indeed, strong family bonds and supportive social networks in Algeria, being comforting for the individual, could act as an antidote to the strain produced by online vigilance; this perhaps depends on one's social environment.

This study contributes to the growing body of literature on digital well‐being by providing novel insights into the mediating role of online and offline social sensitivity. The findings underscore the importance of considering both digital and face‐to‐face social interactions in understanding psychological distress. By focusing on Algerian female students, the study also highlights the cultural and societal factors that shape the relationship between online vigilance and mental health, offering valuable implications for targeted interventions in similar contexts.

### 4.1 Implications of the Findings

This study further develops the social‐cognitive model of internet communication by focusing on the mediating role that social sensitivity plays within the relationship between mental health and online behaviors. We find that online vigilance increases the sensitivity of an individual to social cues, but the way they process and react to these social cues, whether online or offline, is what truly determines their psychological well‐being. The always‐connected nature of these digital platforms seems to amplify social sensitivity, which in turn affects levels of stress, anxiety, and depression.

Furthermore, the differential effects of online and offline social interactions shed more light on digital well‐being. As online vigilance is usually associated with a boost in social sensitivity and largely linked with psychological distress, supportive offline interactions could have some positive effects that may further mitigate such negative effects. This indicates that there is complexity in digital life; while online vigilance does, offline interactions can exert quite opposite influences on mental health.

### 4.2 Cultural Relevance and Practical Implications

These findings have a special relevance for the Algerian context, where societal shifts, coupled with the rapid inroads of digital technologies, have reshaped social interactions. The full mediation observed in this study underlines the importance of addressing online and offline social sensitivity in mental health interventions rather than focusing on online behavior. Cultural norms and societal expectations in Algeria play a significant role in shaping social sensitivity and online vigilance among female students. The emphasis on family harmony, social reputation, and community bonding may heighten the emotional impact of online interactions, particularly in contexts where social rejection or exclusion is perceived as a personal or familial failure. These cultural factors underscore the need for culturally sensitive interventions that address the unique challenges faced by Algerian female students in navigating both online and offline social environments.

### 4.3 Practical Implications

In practice, these findings have several important implications for strategies that mental health interventions may use when targeting female students in Algeria. Balanced interventions need to aim not only at a reduction in online vigilance but also at the fostering of healthy social contacts offline that could counteract the negative consequences of increased social sensitivity. The training programs for the development of social skills need to include strategies for managing online and offline social interactions in such a way that complex social cues are dealt with in a more appropriate manner, especially regarding sensitivity to social rejection. Such training in mindfulness and resilience could enable students to control their reactivity regarding social cues while digital literacy programs teach them how to manage their online presence and balance online interactions to minimize a lot of the stress and anxiety associated with vigilance online. Further, the findings of this study have significant implications for mental health interventions targeting female university students in Algeria. Programs should aim to reduce online vigilance by promoting digital literacy and mindfulness practices, helping students manage their online presence more effectively. At the same time, interventions should foster healthy offline social interactions, providing students with the skills to navigate complex social cues and build supportive relationships. By addressing both online and offline social sensitivity, these programs can mitigate the negative effects of heightened social sensitivity and promote psychological well‐being.

### 4.4 Limitations and Future Directions

Although our study illuminates many valuable insights, some limitations do need to be acknowledged. The cross‐sectional nature of the design inhibits our potential to establish causal relationships, and the focus on female students from Algeria may result in limiting generalizability. It is expected that future studies will investigate these relationships over time through longitudinal designs and involve more diverse populations. This will allow researchers to explore the temporal dynamics of these relationships and confirm the directionality of the observed effects.

Future research might also develop the issue of which aspects of social sensitivity most strongly underpin the relationship between online vigilance and psychological distress. Further, the cultural issues in Algeria influencing online and social sensitivity may go further toward explaining certain very specific difficulties for Algerian female students in managing both their online and offline social worlds.

## 5 Conclusion

Our findings contribute to a deeper understanding of digital well‐being by highlighting the mechanisms at which online vigilance influences stress, anxiety, and depression. Future interventions should facilitate individuals in managing their social sensitivity both online and offline for better psychological health in the amount of support one receives. Further, this study demonstrates that online and offline social sensitivity fully mediates the relationship between online vigilance and psychological distress among Algerian female students. The findings highlight the complex interplay between digital behaviors and social‐cognitive factors in shaping mental health outcomes. By emphasizing the cultural relevance of the results, the study underscores the need for interventions that address both online and offline social sensitivity to promote psychological well‐being. Future research should explore these relationships in diverse populations and over time to further validate these findings and inform culturally sensitive mental health strategies.

## Ethics Statement

All procedures involving human participants were conducted in accordance with the ethical standards of the institutional research committees (Faculty of Humanities and Social Sciences at the University of Chlef and the Director of Education for the province of Chlef, Algeria) and with the 1964 Helsinki Declaration and its later amendments or comparable ethical standards. Ethical approval was obtained from the relevant committees. Informed consent was obtained from all individual participants included in the study.

## Conflicts of Interest

The authors declare no conflicts of interest.

## Data Availability

The datasets generated and analyzed during the current study are available from the corresponding author on reasonable request.
